# ABA INSENSITIVE 2 promotes flowering by inhibiting OST1/ABI5-dependent *FLOWERING LOCUS C* transcription in Arabidopsis

**DOI:** 10.1093/jxb/erae029

**Published:** 2024-01-27

**Authors:** Akhtar Ali, Shah Zareen, Junghoon Park, Haris Ali Khan, Chae Jin Lim, Zein Eddin Bader, Shah Hussain, Woo Sik Chung, Tsanko Gechev, Jose M Pardo, Dae-Jin Yun

**Affiliations:** Institute of Glocal Disease Control, Konkuk University, Seoul 05029, South Korea; Department Molecular Stress Physiology, Center of Plant Systems Biology and Biotechnology, Plovdiv 4000, Bulgaria; Department of Biomedical Science & Engineering, Konkuk University, Seoul 05029, South Korea; Institute of Glocal Disease Control, Konkuk University, Seoul 05029, South Korea; Department of Biomedical Science & Engineering, Konkuk University, Seoul 05029, South Korea; Institute of Glocal Disease Control, Konkuk University, Seoul 05029, South Korea; Department of Biomedical Science & Engineering, Konkuk University, Seoul 05029, South Korea; Division of Applied Life Science, Plant Molecular Biology and Biotechnology Research Center, Gyeongsang National University, Jinju 660-701, South Korea; Division of Applied Life Science, Plant Molecular Biology and Biotechnology Research Center, Gyeongsang National University, Jinju 660-701, South Korea; Department Molecular Stress Physiology, Center of Plant Systems Biology and Biotechnology, Plovdiv 4000, Bulgaria; Department of Plant Physiology and Molecular Biology, Plovdiv University, Plovdiv 4000, Bulgaria; Instituto de Bioquímica Vegetal y Fotosíntesis, cicCartuja, CSIC-Universidad de Sevilla, Americo Vespucio 49, Sevilla-41092, Spain; Department of Biomedical Science & Engineering, Konkuk University, Seoul 05029, South Korea; University College Dublin, Ireland

**Keywords:** ABI2, ABI5, ABA signaling, FLC, flowering time, SnRK2

## Abstract

The plant hormone abscisic acid (ABA) is an important regulator of plant growth and development and plays a crucial role in both biotic and abiotic stress responses. ABA modulates flowering time, but the precise molecular mechanism remains poorly understood. Here we report that ABA INSENSITIVE 2 (ABI2) is the only phosphatase from the ABA-signaling core that positively regulates the transition to flowering in Arabidopsis. Loss-of-function *abi2-2* mutant shows significantly delayed flowering both under long day and short day conditions. Expression of floral repressor genes such as *FLOWERING LOCUS C* (*FLC*) and *CYCLING DOF FACTOR 1* (*CDF1*) was significantly up-regulated in *abi2-2* plants while expression of the flowering promoting genes *FLOWERING LOCUS T* (*FT*) and *SUPPRESSOR OF OVEREXPRESSION OF CONSTANS 1* (*SOC1*) was down-regulated. Through genetic interactions we further found that *ost1-3* and *abi5-1* mutations are epistatic to *abi2-2*, as both of them individually rescued the late flowering phenotype of *abi2-2*. Interestingly, phosphorylation and protein stability of ABA INSENSITIVE 5 (ABI5) were enhanced in *abi2-2* plants suggesting that ABI2 dephosphorylates ABI5, thereby reducing protein stability and the capacity to induce *FLC* expression. Our findings uncovered the unexpected role of ABI2 in promoting flowering by inhibiting ABI5-mediated *FLC* expression in Arabidopsis.

## Teaser

ABI2, an ABA-regulated protein phosphatase, promotes flowering initiation in Arabidopsis through inhibition of a kinase activator of ABI5, the transcription factor regulating the flowering repressor gene *FLC* .

## Introduction

Due to their sessile nature, plants have evolved the ability to alter their physiology and development to adapt to environmental challenges (Bohnert *et al.*, 1995). Unfavorable conditions, like high salinity, cold, or drought stress, are important challenges in agriculture as they reduce the yields of crop plants (Munns *et al.*, 2012). Plant hormones play a key role in environmental acclimation by inducing many biochemical and physiological changes to control both biotic and abiotic stresses (Gray, 2004; Adie *et al.*, 2007; Cao *et al.*, 2011; Finkelstein, 2013; Seo *et al.*, 2014; Pozo *et al.*, 2015). Among them, ABA is an important regulator of plant growth and development and plays a crucial role in both biotic and abiotic stress responses (Lee *et al.*, 2006; Adie *et al.*, 2007; Lee *et al.*, 2009; Mang *et al.*, 2012; Finkelstein, 2013). ABA regulates multiple physiological processes such as seed maturation, embryo morphogenesis, stomatal movement, and floral transition (Yoshida *et al.*, 2002; Finkelstein, 2013; Wang *et al.*, 2013; Murata *et al.*, 2015). However, the mechanism of ABA-delayed flowering time in Arabidopsis is poorly understood.

In the absence of ABA, type 2C Ser/Thr protein phosphatases (PP2C), including ABA INSENSITIVE (ABI) 1 and ABI2, bind to and inhibit kinases of the SNF1-RELATED KINASE2 (SnRK2s) family. The perception of ABA signal is achieved by specific receptors known as PYRABACTIN RESISTANCE (PYR1)/PYR-Like (PYLs)/REGULATORY COMPONENTS OF ABA RESPONSE (RCAR) (Ma *et al.*, 2009; Park *et al.*, 2009). Upon ABA binding, the ABA receptors recruit the PP2C phosphatases, which allows the activation of SnRK2s through autophosphorylation (Fujii *et al.*, 2009; Geiger *et al.*, 2009; Ma *et al.*, 2009; Park *et al.*, 2009; Rodrigues *et al.*, 2013). Activated SnRK2s phosphorylate downstream targets including ABA-RESPONSIVE ELEMENT BINDING FACTORS (ABFs/ABI5) (Fujii *et al.*, 2009; Lee *et al*., 2009; Nakashima *et al.*, 2009). ABFs/ABI5 are members of the bZIP transcription factors, which promote the expression of ABA-responsive genes (Furihata *et al.*, 2006, Fujii *et al.*, 2009; Yoshida *et al.*, 2010; Seo *et al.*, 2014).

In Arabidopsis, ABFs/ABI5 transcription factors are involved in ABA signal transduction during seed germination and/or in vegetative growth (Choi *et al.*, 2000; Jakoby *et al.*, 2002). ABF1, ABF2, ABF3, ABF4, and AREB3 are mainly expressed in vegetative tissues whereas ABI5 is preferentially expressed during seed maturation and seed germination (Finkelstein and Lynch, 2000; Lopez-Molina and Chua, 2000; Uno *et al.*, 2000).

ABI4 and ABI5 are important factors through which ABA inhibits floral transition. ABI5 directly binds to the *FLOWERING LOCUS C* (*FLC*) gene promoter to enhance transcription, with the result of delayed flowering time (Wang *et al.*, 2013). FLC is a major repressor of *FLOWERING LOCUS T* (*FT*) and *SUPRESSOR OF OVEREXPRESSION OF CONSTANS1* (*SOC1*) transcription thereby negatively regulating flowering time (Michaels and Amasino, 1999; Helliwell *et al.*, 2006; Chiang *et al*., 2009). ABI4, an APETELA2/ETHYLENE RESPONSIVE FACTOR (AP2/ERF) domain-containing transcription factor, was also shown to negatively regulate flowering through enhanced *FLC* transcription (Soderman *et al.*, 2000; Shu *et al.*, 2016). SnRK2 kinases, particularly SnRK2.2, SnRK2.3, and SnRK2.6/OPEN STOMATA1 (OST1), which are crucial ABA signaling intermediaries, were also shown to suppress flowering by phosphorylating and activating ABI5 (Wang *et al.*, 2013). These reports indicate that among the ABA signaling components ABI4 and ABI5 specifically control flowering initiation in Arabidopsis and that ABI5 but not ABI4, requires SnRK2-dependent phosphorylation to activate *FLC* transcription (Wang *et al.*, 2013; Shu *et al.*, 2016). Clade A of PP2C protein phosphatases, including ABI1 and ABI2, negatively regulate ABA signaling by inhibiting SnRK2s through dephosphorylation (Merlot *et al.*, 2001; Nishimura *et al.*, 2004; Wasilewska *et al.*, 2008). However, the involvement of PP2Cs (ABI1/ABI2) in regulation of flowering time has remained unknown.

To investigate the role of PP2Cs in flowering, we screened several mutants of the ABA signaling components for flowering phenotypes and found ABI2 to be the only PP2C promoting flowering initiation. Loss-of-function *abi2-2* mutant showed significantly delayed flowering, whereas *abi2-1* (a dominant gain-of-function mutant of *ABI2*) showed WT-like flowering. Expression of genes that suppress flowering, such as *FLC* and CYCLING DOF FACTOR1 (*CDF1*), was up-regulated in *abi2-2* plants whereas expression of *FT* and *SOC1* that promote flowering was down-regulated. Additionally, the phosphorylated status and protein stability of ABI5 was found to be significantly higher in *abi2-2* plants indicating that ABI2 dephosphorylates and thereby reduces ABI5 protein stability, which inhibits FLC transcription. Taken together our results suggest that ABI2 positively regulate flowering by inhibiting OST1- and ABI5-mediated *FLC* transcriptional activation.

## Materials and methods

### Plant materials and growth assay

Arabidopsis ecotype Columbia-0 (Col-0) and Langsberg *erecta* (Ler) were used as wild type (WT) in this study. The *abi1-2*, *abi2-2*, *hab1-1*, *ost1*, *abi5*, *flc-3*, and *pyr1/pyl1/2/4* alleles have been described previously (Michaels and Amasino, 1999; Leonhardt *et al.*, 2004; Saez *et al.*, 2004, 2006; Kuhn *et al.*, 2006; Shu *et al.*, 2016; Yu *et al.*, 2016). Seeds of *abi1-1* and *abi2-1* used in this study were previously described (Leung *et al.*, 1997). To generate the *abi2/ost1*, *abi2/flc-3*, and *abi2/abi5* double mutants, *abi2-2* was crossed with *ost1*, *flc-3*, and *abi5*, respectively. The resulting F_2_ individuals were genotyped by PCR for confirmation of the double mutants. For growth assays, seeds were surface-sterilized and germinated on half-strength Murashige and Skoog (MS) medium supplemented with 0.25% Phytagel (horizontal MS plates) or 1.2% agar (vertical MS plates) and 2% sucrose. For soil-grown plants, we used a commercially available soil, ‘Heungnong bio topsoil for urban farming and potting gardening’ (product no. 398083960). Plants were grown at 23 °C under long-day (16 h light and 8 h dark) or short-day (8 h light and 16 h dark) growth conditions in a controlled culture room with 130 μmol m^−2^ s^−1^ light intensity.

### Vector construction and generation of transgenic plants

For the generation of *ABI2-OX* transgenic plants, the coding sequence of *ABI2* was cloned in the *pDONR/Zeo* GATEWAY vector (Thermo Fisher Scientific, Waltham, MA, USA). This entry vector was further sub-cloned in destination vector, pGWB14 (Thermo Fisher Scientific), and transformed into Col-0 plants using *Agrobacterium* (GV3101)-mediated floral dip method. Primers used for cloning are listed in Supplementary Table S1.

### Generation of *ABI2*-CRISPR lines

Generation of *ABI2-*CRISPR lines was carried out as previously described by Liu *et al*. (2015). ABI2 guide RNAs were designed to avoid complementarity with ABI1. Sequences of ABI2-sgRNAs are listed in Supplementary Table S1. For confirmation of stable mutants, we carried out three different assays: (i) DNA sequencing analysis, (ii) amplification fragment length polymorphism (AFLP), and (iii) qRT-PCR analysis (for detail protocol see Liu *et al.*, 2015). Several lines were selected and among them six T_3_ lines that had reduced *ABI2* expression were used for experiments. Four lines (C-2, C-3, C-4, and C-5) showed deletion of a 918 bp region between two sgRNA sites. One line (C-8) showed a single nucleotide insertion near the first sgRNA site that led to a premature stop codon, while another line (C-10) showed an eight-nucleotide deletion that also led to a premature stop codon near the first sgRNA site.

### RNA isolation, qRT-PCR analysis

Total RNAs extracted from seedlings using the RNeasy Plant Mini Kit (Qiagen, Germantown, MD, USA) and treated with DNase (Sigma-Aldrich, St Louis, MO, USA) were used for the synthesis of first-strand cDNA using the Thermoscript RT-PCR System (Thermo Fisher Scientific). PCR amplification was performed using e-Taq DNA polymerase (Solgent, Daejeon, South Korea). The conditions of real-time PCR were as follows: 95 °C for 5 min, 45 cycles of 95 °C for 10 s and 60 °C for 30 s, followed by 95 °C for 10 s, 65 °C for 5 s, and 95 °C for 5 s. The ΔΔ*C*_t_ method was used for qPCR data analysis. *UBQ5* and *TUB2* were used as reference genes. The primers used in RT-PCR or real-time PCR are listed in Supplementary Table S1.

### Immunoblot analysis

Ten-day-old Arabidopsis plants either treated or untreated with ABA were used for western blot assays. Proteins were extracted and immunoblot analysis was carried out using rabbit polyclonal antibody α-ABI5 (Abcam, cat. no. ab98831) to detect ABI5.

### In-gel kinase assay

An in-gel kinase assay was performed as described before (Kim *et al.*, 2017). Briefly, total proteins were extracted from 10-day-old seedlings. Equal amounts of total plant protein (50 μg) were loaded on 10% SDS-PAGE gel embedded with 0.1 mg ml^−1^ ABI5–glutathione *S*-transferase (GST) substrate. After electrophoresis, SDS was removed by incubating in washing buffer (25 mM Tris–HCl (pH 7.5), 0.5 mM dithiothreitol (DTT), 5 mM NaF, 0.1 mM Na_3_VO_4_, 0.05% BSA, and 0.1% Triton X-100) three times at 22 °C for 1 h each. The gel was renatured at 4 °C with renaturation buffer containing 25 mM Tris–HCl (pH 7.5), 0.5 mM DTT, 5 mM NaF, and 0.1 mM Na_3_VO_4_ with three buffer exchanges for 1, 12, and 1 h. After pre-incubation with 30 ml kinase reaction buffer without ATP at room temperature for 30 min, the gel was incubated in 20 ml kinase reaction buffer (25 mM Tris–HCl (pH 7.5), 2 mM EGTA, 12 mM MgCl_2_, 1 mM DTT, 0.1 mM Na_3_VO_4_, 250 nM ATP, and 50 μCi [γ-^32^P]ATP) for 1.5 h. The gel was washed with stop buffer [5% trichloroacetic acid (w/v) and 1% potassium pyrophosphate (w/v)] for 5 h at room temperature with five buffer exchanges. The gel was dried on 3M paper and imaged using a Fujifilm FLA-5000 imaging system.

## Results

### Loss-of-function *abi2-2* mutation delays flowering time

**Fig. 1.**
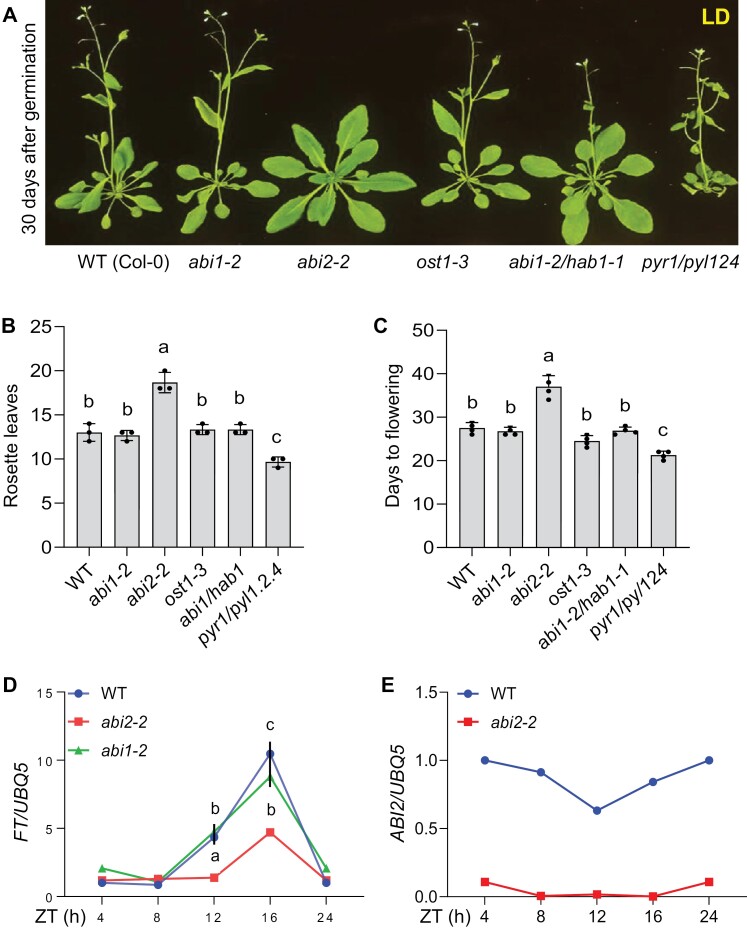
Flowering phenotype of ABA-signaling mutants. The flowering time was analysed under long-day (LD) growth conditions. (A) Representative images of 30-day-old soil-grown WT (Col-0), *abi1-2*, *abi2-2*, *ost1-3*, *ab1-2/hab1-1*, and *pyr1/pyl1/2/4* plants under LD growth conditions. (B, C) Flowering time scored as the number of rosette leaves at flowering (B) and number of days from germination to flowering (C) of WT and indicated genotypes under LD growth conditions. Error bars represent SE from three independent biological repeats (*n*=8 in each repeat). Different letters indicate significant difference determined by one-way ANOVA with Bonferroni’s correction (*P*<0.05). (D) Expression analysis of the flowering-time-related marker gene *FT* in WT, *abi1-2*, and *abi2-2* genotypes under LD conditions. *FT* expression was analysed by qRT-PCR. *UBQ5* was used as internal control. Error bars represent SE from three biological replicates. Different letters indicate significant difference determined by one-way ANOVA with Bonferroni’s correction (*P*<0.05). (E) Expression analysis of *ABI2* over a 24 h time course in WT seedlings grown under LD growth condition. *ABI2* expression was analysed by qRT-PCR. *UBQ5* was used as internal control. Error bars represent SE from two biological replicates. Primers used in the qRT-PCR assays are listed in Supplementary Table S1.

**Fig. 2.**
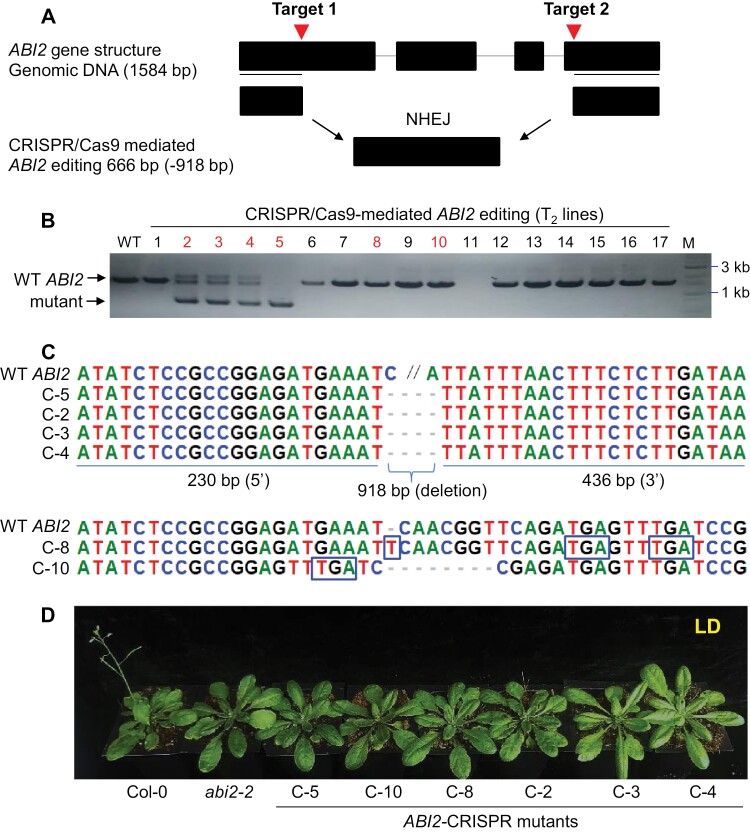
CRISPR/Cas9-mediated *ABI2* mutation delays flowering. (A) Schematic diagram of *ABI2* gene with two sgRNA sites as indicated by target 1 and target 2. After deletion of the middle region between two sgRNA sites through non-homologous end joining DNA repair system, the two sites will bind together and as a result a small sized non-functional ABI2 will be generated. (B) PCR analysis of CRISPR/Cas9 *abi2* lines using genomic DNA. Lines 2, 3, 4, and 5 showed deletion of a large portion of *ABI2* gene between two sgRNA sites that resulted in a small band of 666 bp, a non-functional *ABI2* gene. (C) Confirmation of *ABI2* gene editing using DNA sequencing analysis. Lines C-2, C-3, C-4, and C-5 showed deletion of a 918 bp region between two sgRNA sites. Line C-8 showed a single nucleotide insertion while C-10 showed deletion of eight nucleotides near first sgRNA site in *ABI2* gene that resulted in a premature stop codon. (D) CRISPR/Cas9-mediated *ABI2* mutation delays flowering. Representative (30-day-old) soil-grown WT, *abi2-2*, and *ABI2*-CRISPR mutant lines (C-5, C-10, C-8, C-2, C-3, and C-4) under LD conditions (with three biological replicates, *n*=8 in each repeat). For statistical analysis see Supplementary Fig S1.

To assess the involvement of ABA signaling in floral transition, the flowering time phenotypes of several ABA signaling mutants were analysed under long-day (LD) conditions. Only the *abi2-2* mutant among all tested genotypes showed late flowering, as indicated by the number of rosette leaves and the days to flowering (Fig. 1A–C). Other tested mutants showed either a similar phenotype to that of WT (Col-0) or slightly early flowering (*pyr1/pyl1*,*2*,*4*) (Fig. 1A–C). To further confirm the late flowering phenotypes of *abi2-2*, we tested the expression of *FT*, a flowering marker gene, in the *abi2-2* knockout mutant (Turck *et al.*, 2008). The transcript level of *FT* began to accumulate at ZT12 (zeitgeber time) and peaked at ZT16 both in WT and *abi1-2* plants. However, consistent with late flowering phenotypes, the expression of *FT* was also less induced in *abi2-2* (Fig. 1D). Interestingly, the transcript level of *ABI2* over the 24 h circadian loop under LD condition showed a slight decrease in the evening (ZT12–ZT16) (Fig. 1E). To corroborate results with the *abi2-2* allele, more loss-of-function *abi2* alleles were generated using CRISPR. Several *ABI2*-CRISPR mutants were analysed for *ABI2* gene editing using genotyping and DNA sequencing (Fig. 2A–C). Among them four lines showed a 918 bp deletion (C-2, C-3, C-4, C-5), one line (C-8) showed a single nucleotide insertion that resulted in a premature stop codon, and another line (C-10) showed a sequence rearrangement that also produced a premature stop codon (Fig. 2C). Moreover, the transcript level of *ABI2* was dramatically reduced in all these lines (Supplementary Fig. S1A). When tested for flowering phenotypes, like *abi2-2*, *ABI2*-CRISPR mutants also showed late flowering phenotype as compared with WT (Fig. 2D; Supplementary Fig. S1B, C). Taken together these results demonstrate that ABI2 is a major positive regulator of the ﬂoral transition in Arabidopsis, whose deficiency results in delayed flowering.

### Overexpression of *ABI2* does not accelerate flowering under LD growth condition

**Fig. 3.**
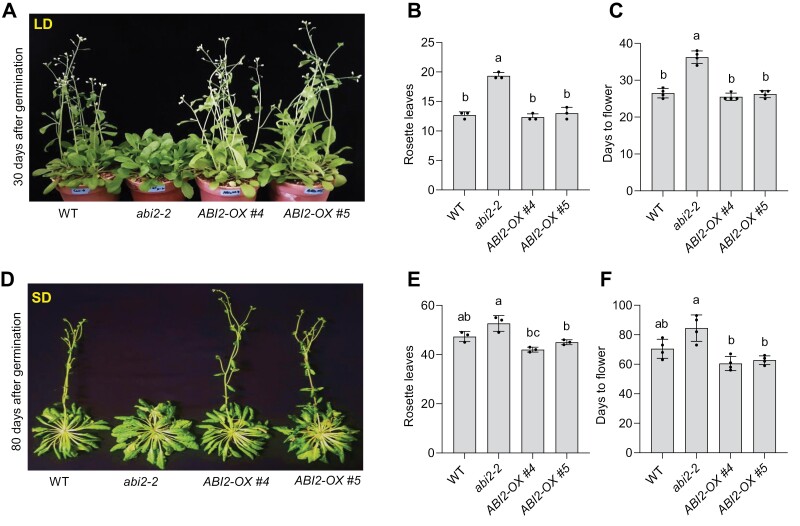
Flowering phenotypes of ABI2 knockout mutant and overexpressing plants under long-day (LD) and short-day (SD) growth conditions. (A) Representative 30-day-old soil-grown plants of WT, *abi2-2*, and two transgenic lines overexpressing *ABI2* under *35S* promoter (OX #4 and OX #5), under LD growth conditions. (B) Flowering time scored as the number of rosette leaves at flowering. (C) Flowering time scored as the days from germination. (D) Representative (80-day-old) soil-grown WT, *abi2-2*, and overexpression lines (OX #4 and OX #5) under SD growth conditions. (E) Flowering time scored as the number of rosette leaves at flowering of WT, *abi2-2* and overexpression lines (OX #4, OX #5) under SD growth conditions. (F) Flowering time scored as days from germination to flowering of the indicated genotypes under SD growth conditions. In all plots, error bars represent SE from three independent biological repeats (*n*=8 in each repeat). Different letters indicate significant difference determined by one-way ANOVA with Bonferroni’s correction (*P*<0.05).

Since *abi2-2* knockout and *ABI2*-CRISPR mutants showed late flowering, we were interested to test the flowering phenotypes of ABI2-overexpression lines. We generated Arabidopsis transgenic plants overexpressing ABI2 under the *35S* promoter to test promotion of flowering (Supplementary Fig. S2). Overexpression of *ABI2* (*ABI2-OX #4* and *ABI2-OX #5*) did not show any pronounced early-flowering phenotype under LD condition (Fig. 3A–C). Next, we tested flowering time under short day (SD) conditions. Consistent with LD conditions, *abi2-2* mutant also delayed flowering under SD (Fig. 3D–F). Interestingly, *ABI2-OX #4* and *ABI2-OX #5* showed early flowering phenotypes under SD conditions compared with that of WT plants (Fig. 3D–F).

Previous reports have shown that the *abi2-1* mutation causes a substitution of Gly by Asp (G168D) in the phosphatase catalytic domain, which confers gain-of-function and dominant ABA-insensitivity by unconditional inhibition of SnRK2s activity (Leung *et al.*, 1997; Umezawa *et al.*, 2009; Vlad *et al.*, 2009). To test whether the dominant phosphatase mutant *abi2-1* also affected flowering time, we analysed the flowering phenotypes of *abi2-1* together with *abi2-2* and *abi1-2* as controls. As expected, *abi2-2* plants showed late flowering while *abi1-2* phenotypes were similar to those of WT (Supplementary Fig. S3A–C). By contrast, the *abi2-1* dominant mutant showed WT-like (Ler) flowering time, as indicated by the number of rosette leaves and days to flowering (Supplementary Fig. S3A–C). Taken together, these findings suggest that ABI2 positively regulate flowering initiation in Arabidopsis.

### *ABI2* mutation alters the expression pattern of flowering-related genes

**Fig. 4.**
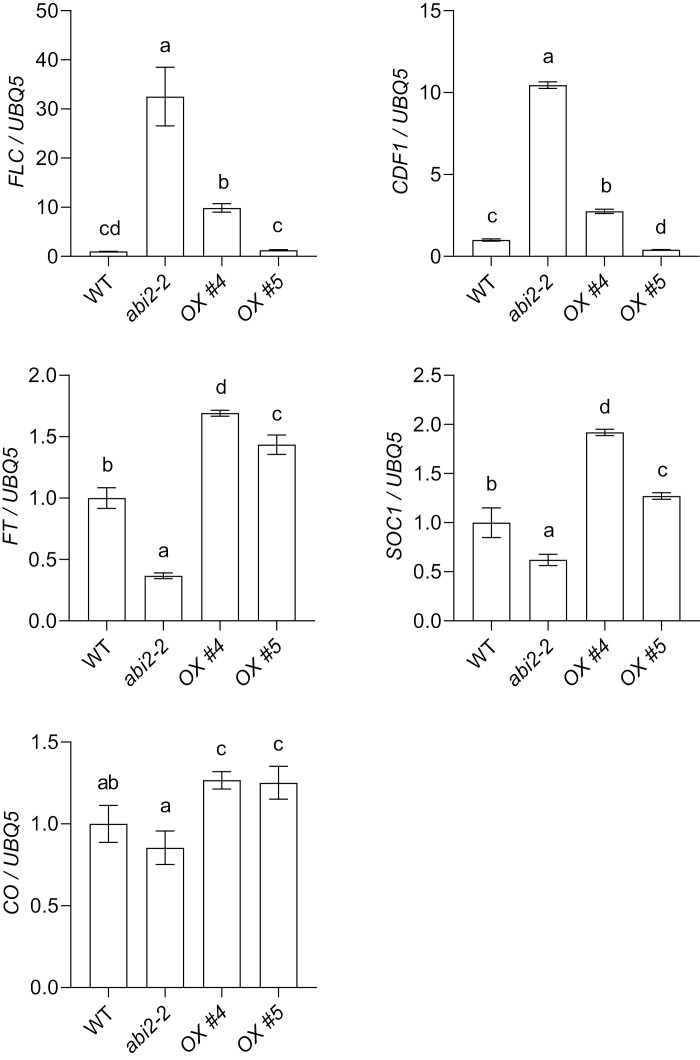
Expression of flowering-related genes. Expression analysis of the flowering-time-related marker genes *FLC*, *CDF1*, *FT*, *SOC1*, and *CO* in WT, *abi2-2*, and *ABI2*-overexpressing lines (OX #4 and OX #5) under LD conditions. Transcript level of *FLC* and *CDF1* increased in *abi2-2* plants whereas that of *FT* and *SOC1* was significantly decreased in *abi2-2* mutant. Transcript level was analysed by qRT-PCR. *UBQ5* was used as internal control. Error bars represent SE from three biological repeats. Different letters indicate significant difference determined by one-way ANOVA with Bonferroni’s correction (*P*<0.05). Primers used in the qRT-PCR assay are listed in Supplementary Table S1.

To further explore the molecular mechanisms through which ABI2 controls floral transition, the transcript levels of genes that regulate flowering time were analysed in the *abi2-2* mutant and *ABI2* overexpressing plants (*ABI2-OX*). FLC directly represses the flowering identity genes *FT* and *SOC1* thereby delaying flowering time in Arabidopsis (Helliwell *et al.*, 2006; Searle *et al.*, 2006; Deal *et al.*, 2007). To explore whether the flowering phenotypes observed for the *abi2-2* mutant and *ABI2-OX* correlate with the change of *FLC* expression and its downstream regulon, we examined the *FLC* transcript levels in these genotypes by qRT-PCR. The level of *FLC* transcript in the *abi2-2* mutant plants was induced up to 8-fold, while in *ABI2-OX* plants it was similar to that in the WT plants (Fig. 4; Supplementary Fig. S4B). Furthermore, the expression of *FT* and *SOC1* was significantly decreased in the *abi2-2* mutant (Fig. 4; Supplementary Figs S4A, S5). The expression of *CONSTANS* (*CO*), whose protein is the activator of *FT* and *SOC1* expression, was not altered in *abi2-2* plants, but it was marginally increased in *ABI2-OX* lines (Fig. 4). The transcript level of *FLC* was also up-regulated while that of *FT* was down-regulated in ABI2 CRISPR mutants (Supplementary Fig. S6). Interestingly, the transcript level of *CDF1*, whose protein negatively regulates *FT* and *CO*, was also increased in *abi2-2* mutant (Fig. 4; Supplementary Fig. S4B). These changes in the transcript level of flowering-related genes were consistent with the flowering phenotype, suggesting that the late flowering phenotype of *abi2-2* may result from FLC-dependent *FT* and *SOC1* repression.

### ABI2 reduces ABI5 protein stability through dephosphorylation

**Fig. 5.**
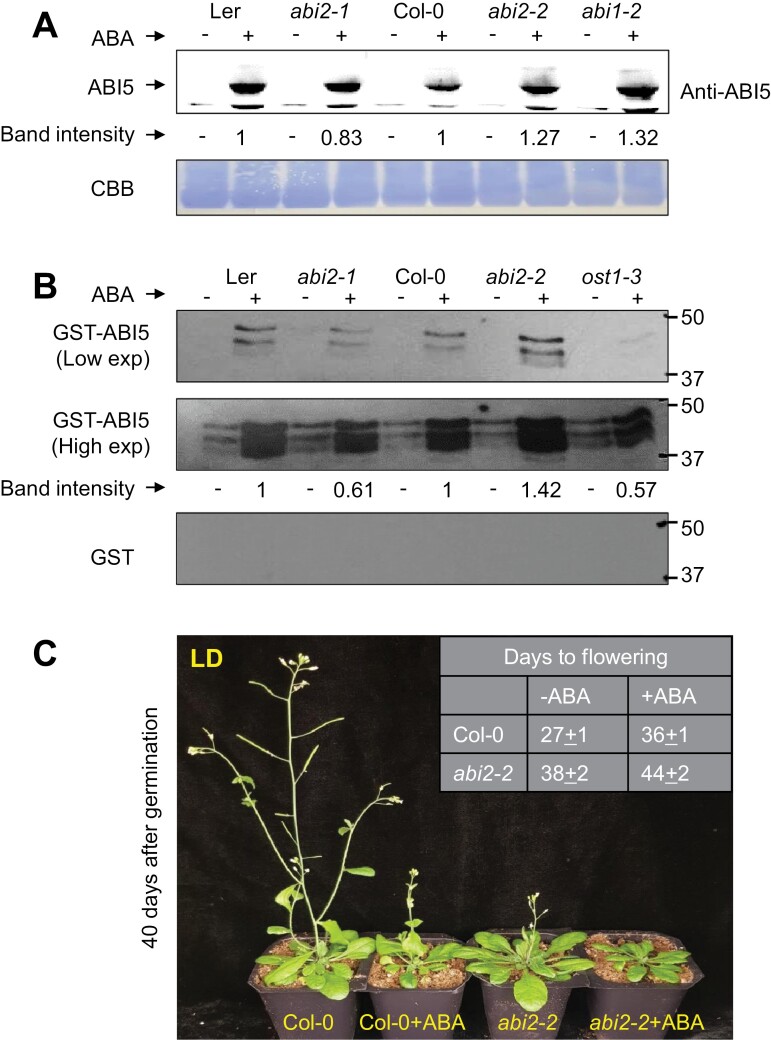
ABI2 suppresses ABI5 protein stability through dephosphorylation. (A) ABI2-mediated destabilization of ABI5 protein. Ten-day-old MS plate-grown seedlings of indicated genotypes were treated with 100 μM ABA (+) or mock-treated without (−) for 3 h and then total proteins were extracted. Western blot was carried out using anti-ABI5 antibodies. Relative band intensity of ABI5 for each mutant allele was compared with its corresponding WT (*abi2-1* compared with Ler; *abi2-2*, *abi1-2*, and *ost1* were compared with Col-0). CBB indicates equal amount of protein loading. The experiment was repeated three times with similar results. (B) In-gel kinase assay using ABI5–GST as substrate. Signal intensity of in-gel kinase assay on phosphorylating the ABI5–GST after treatment without (−) or with (+) ABA (100 μM) for 3 h. The ABA-induced bands represent the activated SnRK2s. Relative radioactivity intensity of ABA-inducible bands are indicated as band intensity (only ABA-induced bands were quantified). Each allele was compared with its corresponding WT (*abi2-1* was compared with Ler whereas *abi2-2* and *ost1-3* were compared with Col-0). GST alone was used as negative control. Experiment was repeated twice with similar results. (C) Application of exogenous ABA delayed flowering. Three-week-old soil-grown plants were sprayed with mock control (water) (−ABA) or with 100 µM ABA (+ABA) twice a week for 2 weeks. Photographs were taken after 18 d after the first treatment. The experiment was repeated three times with similar results (*n*=8 in each repeat).

ABI5 directly binds to the promoter of *FLC* and positively regulates its transcription (Wang *et al.*, 2013). To test whether *abi2-2* also affects *ABI5* transcription, we determined the transcript level of *ABI5* in *abi2-2* mutant and *ABI2-OX* plants. The expression of *ABI5* gene was only slightly induced in *abi2-2* plants (*P*=0.099, *t*-test) and was not altered in *ABI2-OX* plants (Supplementary Fig. S7A), indicating that ABI2 might affect ABI5 post-transcriptionally. To test this hypothesis, we investigated the protein level of ABI5 in *abi2-2* knockout and *abi2-1* dominant phosphatase mutant plants. Notably, the ABI5 protein level was highly accumulated in *abi2-2* plants upon ABA treatment (Fig. 5A; Supplementary Fig. S7B, C). Moreover, ABI1, a homolog of ABI2, also affected ABI5 stability as described by the high amount of ABI5 protein in *abi1-2* mutant (Fig. 5A). Consistent with transcription data, the ABI5 protein level in *ABI2*-overexpression was also the same as that in WT (Supplementary Fig. S4B, C). By contrast, ABI5 was less accumulated in the phosphatase dominant mutant, *abi2-1*, compared with the congenic WT (Ler) (Fig. 5A). Taken together, these results suggest that ABI2 reduces ABI5 stability. Next, we carried out an in-gel kinase assay using ABI5–GST as a substrate. Interestingly, the ABA-induced ABI5 phosphorylation was stronger in *abi2-2* mutant and significantly weaker in *abi2-1* dominant mutant than that in their corresponding wild types (Fig. 5B). Furthermore, ABA-mediated ABI5 phosphorylation was much reduced in *ost1-3* plants, indicating that ABI5 phosphorylation was largely due to OST1 activity (Fig. 5B). Taken together, these findings suggest that ABI2 counteracts ABI5 phosphorylation by OST1 and thereby inhibits ABI5-mediated *FLC* expression.

Previously, ABI5 was shown to inhibit floral transition through *FLC* activation in an ABA-dependent manner (Wang *et al.*, 2013). Since ABI5 was accumulated in *abi2-2*, next we tested whether direct application of exogenous ABA modulated flowering. As shown in Fig. 5C, application of ABA delayed flowering for about 9 d in WT (Col-0). In the presence of ABA, flowering time was extended from the 27th day after germination to the 36th day. Mutant *abi2-2* flowered at the 38th day under regular conditions and ABA further delayed flowering by six additional days (Fig. 5C). Taken together, these findings suggest that in the presence of ABA, ABI2 is inhibited and the release of OST1 not only activates ABA signaling but also inhibits flowering by activating the ABI5-FLC module.

### *ost1* and *abi5* mutations suppress the late flowering phenotypes of *abi2-2*

**Fig. 6.**
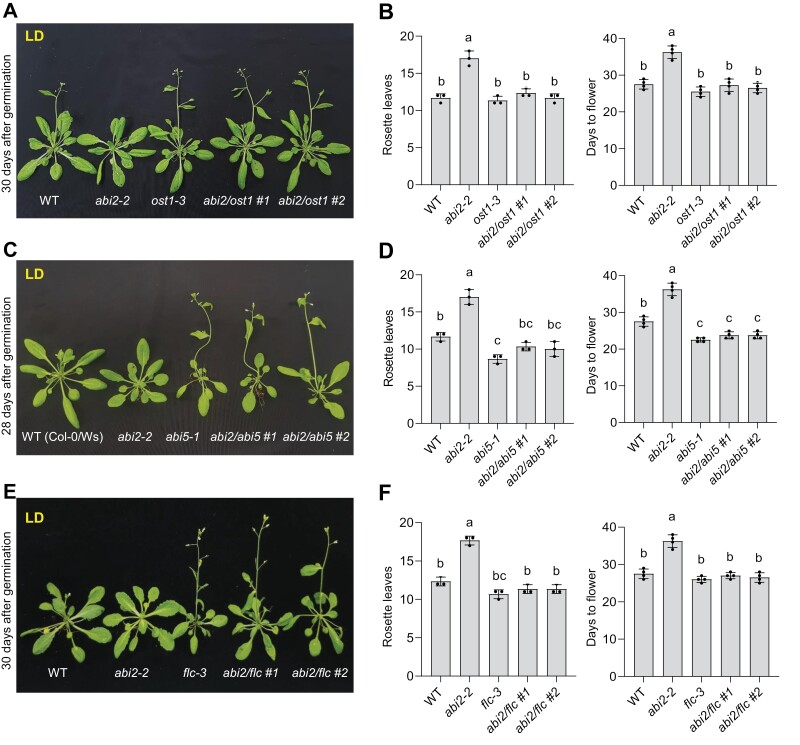
Late-flowering phenotype of *abi2-2* depends on OST1 and ABI5 proteins. Double mutants of *abi2-2* with *ost1-3*, *abi5*, and *flc-3* were generated by genetic crossing, and then the flowering time of these genotypes was examined. (A) Thirty-day-old soil-grown WT, *abi2-2*, *ost1*, and *abi2/ost1* double mutants under LD growth conditions. (B) Flowering time scored as the number of rosette leaves at flowering and the number of days from germination to flowering of WT, *abi2-2*, *ost1-3*, and *abi2/ost1* genotypes under LD growth conditions. (C) Twenty-eight-day-old WT (Col-0/Ws), *abi2-2*, *abi5-1*, and *abi2/abi5* double mutants grown under LD growth conditions. The Col-0/Ws hybrid was used as a wild type control (WT), because *abi2-2* is in the Col-0 background, whereas *abi5-1* is in the Ws background. (D) Flowering time scored as the number of rosette leaves at flowering and the number of days from germination to flowering of WT, *abi2-2*, *abi5-1*, and *abi2/abi5* genotypes under LD growth conditions. (E) Thirty-day-old WT, *abi2-2*, *flc-3* and *abi2/flc-3* double mutants grown under LD growth conditions. (F) Flowering time scored as the number of rosette leaves at flowering and the number of days from germination to flowering of WT, and representative single and double mutant genotypes of *abi2-2* and *flc-3* under LD growth conditions. In all plots, error bars represent SE from three independent biological repeats (*n*=8 in each repeat). Different letters indicate significant difference determined by one-way ANOVA with Bonferroni’s correction (*P*<0.05).

ABI5 requires phosphorylation by OST1 to activate *FLC* transcription (Wang *et al.*, 2013). The late ﬂowering phenotype of *abi2-2* mutant suggested that ABI2 could be a positive regulator of the ﬂoral transition in Arabidopsis, presumably by counteracting OST1- and ABI5-dependent *FLC* activation. Since *FLC* transcript was induced in *abi2-2* plants, we tested whether *abi2-2* late ﬂowering phenotype was due to OST1- and/or ABI5-mediated enhancement of *FLC* expression. For this, we made genetic crosses to obtain a double mutant of *abi2-2* with *ost1-3* and *abi5-1* and tested their ﬂowering phenotypes. Both *abi2/ost1* and *abi2/abi5* double mutant plants completely rescued *abi2-2* late flowering as indicated by the days to flowering and the number of rosette leaves (Fig. 6A–D). Additionally, the transcript level of *FLC* was down-regulated in *abi2/ost1* and *abi2/abi5* double mutant plants indicating that the highly induced *FLC* transcript in *abi2-2* was due to the combined activity of OST1 and ABI5 (Fig. 4; Supplementary Fig. S8A, B). To further confirm this finding, *abi2-2/ﬂc-3* double mutant plants were generated by genetic cross and analysed for flowering phenotypes. As expected, the late flowering of *abi2-2* was completely rescued in the *abi2-2/flc-3* double mutant, as indicated by the number of rosette leaves and by the days to flowering (Fig. 6E, F). It has been shown earlier that the flowering time of *flc-3* is similar to that of WT under LD, but it flowers much earlier than the WT under SD condition (Michael and Amasino, 2001). We therefore tested flowering phenotypes of the *abi2-2/flc-3* double mutant under SD condition. As expected *flc-3* showed early flowering whereas *abi2-2* presented late flowering compared with WT under SD (Supplementary Fig. S9). Interestingly, the *abi2-2/flc-3* double mutant showed earlier flowering than WT under SD suggesting that the *flc-3* mutation is epistatic to *abi2-2* (Supplementary Fig. S9). Altogether, these results show that ABI2 positively regulates flowering through inhibition of OST1- and ABI5-mediated *FLC* expression.

## Discussion

### Involvement of ABA in floral transition

**Fig. 7.**
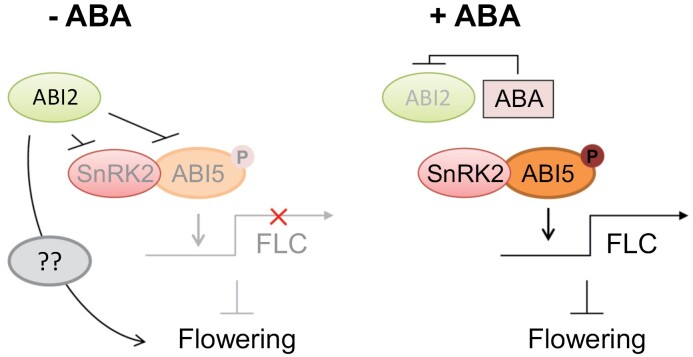
Hypothetical model of the study. A proposed working model. Under regular conditions (−ABA), ABI2 inhibits SnRK2- and ABI5-mediated *FLC* activation thereby promoting flowering. By contrast, ABA inhibits ABI2 thereby releasing SnRK2s, which activate ABI5 through phosphorylation. Activated ABI5 then accumulates and directly activates *FLC* transcription to repress floral transition.

Phytohormones have diverse roles in the growth and development of plants, and regulate multiple physiological, metabolic, and cellular processes, including the floral transition (Gray, 2004; Davis, 2009; Domagalska *et al.*, 2010). Among them, gibberellin (GA) plays a major role in regulating flowering time in the model plant Arabidopsis (Bao *et al.*, 2020; Fukazawa *et al.*, 2021; Zhang *et al.*, 2023). In contrast to GA, the detailed mechanisms by which ABA affects plant flowering are less explored. Drought-induced ABA triggers the drought escape response by promoting FT expression, whereas inhibiting ABA signaling reduces FT expression markedly, highlighting the positive role of ABA in flowering initiation (Riboni *et al.* 2013, 2016). However, another report stated that ABA negatively regulates flowering time by activating an SnRK2s-mediated ABI5–FLC module, a flowering repressor pathway (Wang *et al.*, 2013). The negative role of ABA in floral transition was further supported by the identification of ABI4 as the transcription activator of *FLC* (Shu *et al.*, 2016). These reports suggest that ABA regulates flowering time either positively or negatively. Therefore, the specific details regarding the mechanisms by which ABA signaling modulates plant floral transition needed detailed investigation. Using phenotypic, genetic, and biochemical analysis, we demonstrated that ABI2, a clade A PP2C that negatively regulates SnRK2s and ABA-related transcription factors (Fujii *et al.*, 2009; Vlad *et al.*, 2009; Soon *et al.*, 2012), also promotes floral transition in Arabidopsis through inhibition of ABI5-mediated *FLC* activation (Fig. 7). The late flowering phenotype of *abi2-2* is coherent with the previous finding of the negative affect of ABA on floral transition (Figs 1–3; Wang *et al.*, 2013).

Several ABA-signaling mutants have been demonstrated that possess flowering time phenotypes; for instance, *abf3/abf4* and *abf2/abf3/abf4* show late flowering, whereas *abi4*, *abi5*, and *snrk2s* present early flowering phenotypes (Fujii and Zhu, 2009; Wang *et al.*, 2013; Shu *et al.*, 2016; Hwang *et al.*, 2019). Our findings of ABI2’s involvement in promoting flowering provide yet another piece of strong evidence for the negative effect of ABA signaling on flowering time in plants.

### ABI2 promotes flowering by inhibiting the ABI5–FLC module

Among the 10 members of the SnRK2 family in Arabidopsis, SnRK2.2, SnRK2.3, and SnRK2.6/OST1 regulate vegetative and reproductive growth and also control floral transition (Hrabak *et al.* 2003; Fujii and Zhu, 2009; Nakashima *et al.*, 2009; Wang *et al.*, 2013). The triple mutant of these three SnRK2 kinases (snrk2.2/2.3/2.6) showed reduced phosphorylation of ABFs and ABI5 transcription factors (Nakashima *et al.*, 2009). More importantly, ABI5-mediated activation of the *FLC* promoter also requires ABA-activated SnRK2s (Wang *et al.*, 2013). On the other hand, ABI4 does not require ABA or phosphorylation by SnRK2s to activate *FLC* (Shu *et al.*, 2016), which suggests that interference of ABA with flowering time only occurs through the SnRK2–ABI5 regulatory pathway, while ABI4 might only contribute to ABI5 activation (Bossi *et al.*, 2009; Reeves *et al.*, 2011). Interestingly, more than half of ABI5’s target genes were down-regulated in *snrk2s* triple mutant plants, suggesting that the expression pattern of ABI5-regulated genes largely depend on SnRK2s-mediated ABI5 phosphorylation (Nakashima *et al.*, 2009). The present study shows that ABI5 was more phosphorylated and stabilized in loss-of-function *abi2-2* mutant plants, and less phosphorylated and more destabilized in the *abi2-1* dominant phosphatase mutant (Fig. 5). These findings are coherent with the notion that ABI2 dephosphorylates ABI5 to inhibit the ABI5–FLC module and thereby positively regulate flowering initiation (Fig. 5). Furthermore, *ost1* (*snrk2.6*) mutation recues the *abi2-2* late flowering phenotype, suggesting that the late flowering of *abi2-2* was largely due to enhanced activity of OST1, which is required for the ABI5/FLC module to inhibit flowering initiation (Fig. 6A-C; Fujii and Zhu, 2009; Wang *et al.*, 2013). Additionally, the transcript level of *FLC* was also found to be significantly higher in *abi2-2* mutant (Fig. 4), which correlated with ABI5 stability (Fig. 5). Previous reports have shown that ABI2 interacts with and dephosphorylates SnRK2s, which affects their activity with targets such as ABI5 (Umezawa *et al.*, 2009; Vlad *et al.*, 2009). Consistent with previous findings, ABI5, as one of the major substrates of SnRK2s, was strongly phosphorylated and accumulated in *abi2-2* plants (Fig. 5A, B) which results in strong activation of *FLC* (Fig. 4) and thereby delays flowering.

### ABI2 plays a critical role in the crosstalk between ABA signaling and floral transition

When plants are challenged by environmental stresses such as drought and salt, endogenous ABA level rises and protects plants against the changing environment. However, increased ABA content also affects floral transition, either positively (drought escape in case of severe stress) or negatively (mild stress), as proposed by Shu *et al.* (2018). In the presence of ABA, SnRK2-dependent phosphorylation and activation of ABI5 triggers the expression of *FLC* and results in late flowering (Wang *et al.*, 2013). In this study we investigated how plants deactivate the SnRK2-accelerated ABI5–FLC module to promote normal growth and development, including flowering initiation. We identified ABI2 as the inhibitor of OST1 and ABI5-mediated FLC transcription and presumed that ABI2 is one of the major switches between ABA pathway and flowering time, which not only inhibits ABA signaling but also promotes flowering by negatively regulating ABI5–FLC module.

Recently, we have shown that HOS15-mediated OST1 degradation is required for the desensitization of the ABA signaling cascade (Ali and Yun, 2020). Furthermore, the dephosphorylated form of OST1 was found to be the preferred target of HOS15. For instance, OST1 was more stable in *abi1-2* and *abi2-2* knockout plants but destabilized in the *abi1-1* dominant phosphatase mutant (Ali *et al.*, 2019). Since ABI5 is phosphorylated by SnRK2 kinases, while ABI2 (and other PP2Cs) dephosphorylates SnRK2s, we propose that the increased phosphorylation and protein content levels of ABI5 in the *abi2-2* mutant highlights the ABI2-mediated desensitization of an SnRK2–ABI5–FLC module to promote floral transition. In summary, our findings provide novel insights about the mechanisms that enable plant floral transition by ABI2 through dephosphorylation of ABI5 and subsequent inhibition of the ABI5–FLC module. Understanding how ABI2 is activated to trigger dephosphorylation of ABI5 and/or what other unknown interactors or substrates of ABI2 in the flowering pathway are represents a major goal for future studies.

## Conclusions

The environmentally controlled ABA content provides regulatory flexibility on floral transition through an SnRK2s-activated ABF5–FLC module. Under favorable growth conditions, plants might need the immediate adjustment of ABI5-activated *FLC* expression by ABI2 to properly regulate flowering time. As supported by the double mutant phenotypes of *abi2/ost1*, *abi2/abi5*, and *abi2/flc* (Fig. 6), ABI2 functions as a major flowering regulator by inhibiting SnRK2s (OST1), ABI5 phosphorylation, and indirectly reducing *FLC* transcription.

## Supplementary data

The following supplementary data are available at *JXB* online.

Fig. S1. *ABI2*-CRISPR mutants show late flowering phenotype.

Fig. S2. Transcript level of ABI2 in *ABI2-OX* lines.

Fig. S3. Flowering phenotype of *abi2-1* under LD growth conditions.

Fig. S4. Expression of pattern of flowering-related genes.

Fig. S5. *FT* and *SOC1* were less expressed in *abi2-2* plants.

Fig. S6. Expression pattern of *FLC* and *FT* in *ABI2-CRISPR* lines.

Fig. S7. Transcript and protein levels of *ABI5* in *abi2-2* and *ABI2-OX* plants.

Fig. S8. Transcript level of *FLC* in *abi2/ost1* and *abi2/abi5* double mutants.

Fig. S9. *FLC*-mutation rescues *abi2-2* late flowering phenotypes under SD.

Table S1. Primers used for qRT-PCR, cloning, and sgRNAs (for CRISPR).

erae029_suppl_Supplementary_Figures_S1-S9_Table_S1

## Data Availability

All data supporting the findings of the study are available within the paper and within its supplementary data published online.
