# Supplementary material for: ABA INSENSITIVE 2 promotes flowering by inhibiting OST1/ABI5-dependent FLOWERING LOCUS C transcription in Arabidopsis
Source: J Exp Bot. 2024 Jan 27;75(8):2481–93. doi: 10.1093/jxb/erae029 (PMC11016836; doi:10.1093/jxb/erae029)
Supplement: erae029_suppl_Supplementary_Figures_S1-S9_Table_S1 [file erae029_suppl_supplementary_figures_s1-s9_table_s1.pdf]

Supplementary files

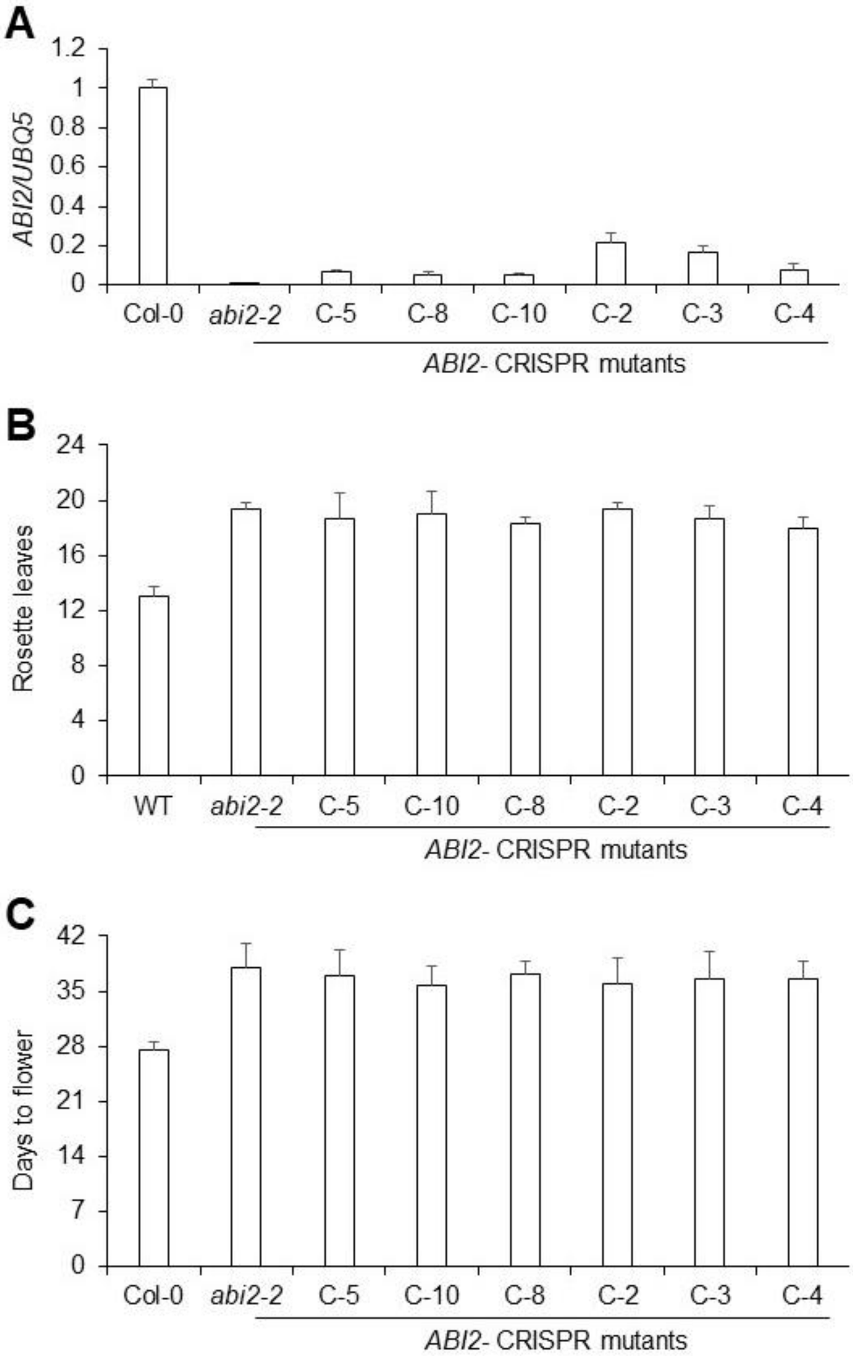

Supplementary Fig. S1. *ABI2*-CRISPR mutants show late flowering phenotype. Late-flowering phenotype of ABI2-CRISPR mutants under long-day (LD) growth conditions. **(A)** Expression analysis of *ABI2* in CRISPR lines using qRT-PCR with primers listed in

Supplementary Table S1. *UBQ5* was used as internal control for normalization. Error bars represent SE from three biological repeats. **(B, C)** Flowering time scored as the number of rosette leaves **(B)** and days from germination to flowering **(C)** of WT, *abi2-2* and *ABI2*-CRISPR mutant lines grown under LD growth conditions in soil. Error bars represent SE from three independent biological repeats (n = 8 in each repeat).

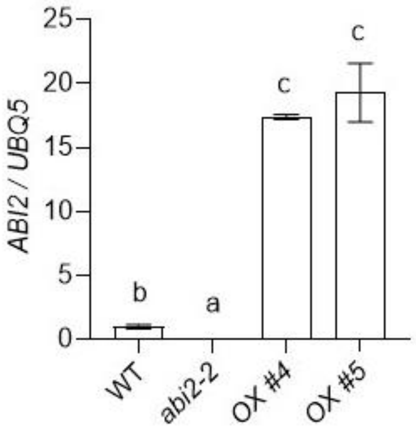

Supplementary Fig. S2. Transcript level of ABI2 in *ABI2-OX* lines. Expression analysis of *ABI2* in 10-day-old seedlings of WT, *abi2-2* and *ABI2-OX* grown on MS plates under LD growth conditions. Transcript level was analyzed by qRT-PCR, and three replications were performed. *UBQ5* was used as internal control. Error bars represent SE from three biological replicates. Different letters indicate significant difference determined by one-way ANOVA test with Bonferroni's correction ($p<0.05$). Primers used in the qRT-PCR assay are listed in Supplementary Table S1.

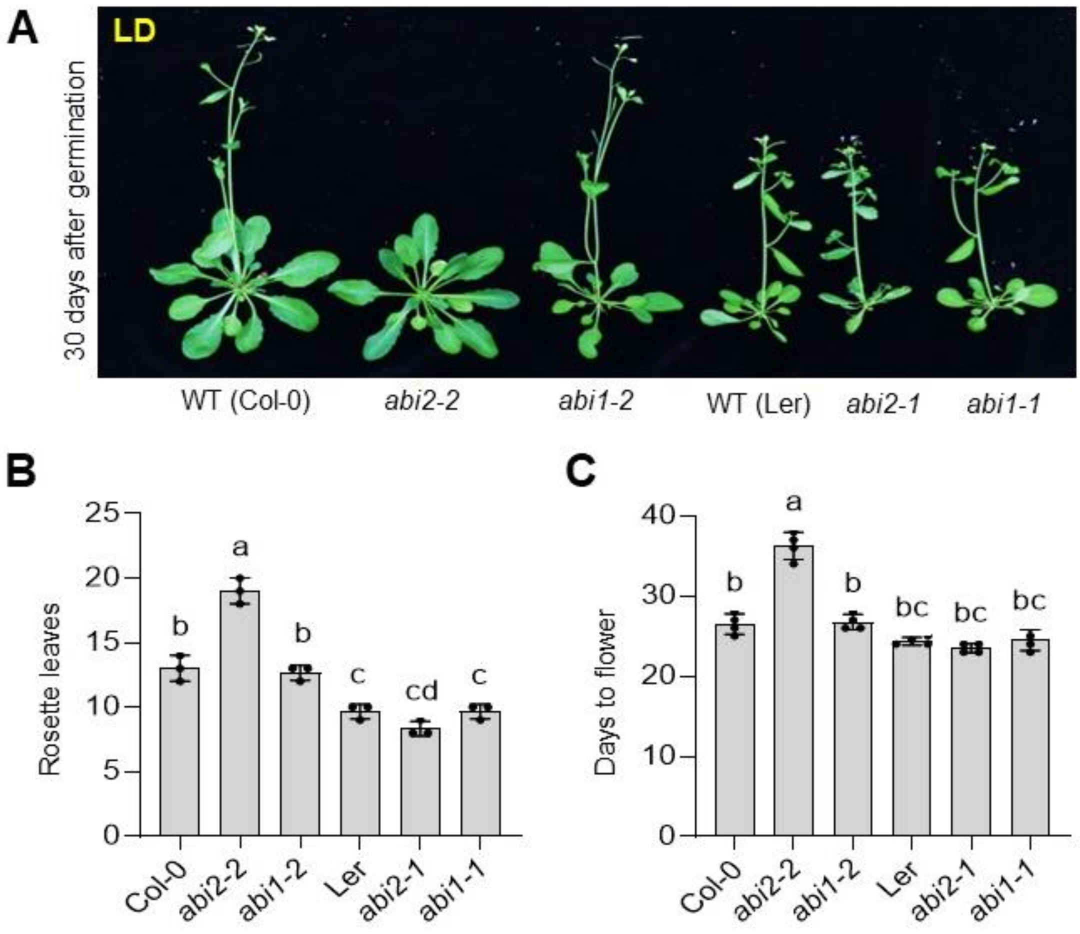

Supplementary Fig. S3. Flowering phenotype of *abi2-1* under LD growth conditions. (A) Representative images of 30-day-old soil grown Col-0 (WT), *abi1-2* and *abi2-2*, and Ler (WT), *abi2-1* and *abi1-1* plants under LD conditions. **(B)** Flowering time scored as the number of rosette leaves at flowering of the indicated genotypes under LD growth conditions. **(C)** Flowering time scored as the days from germination to flowering of the indicated genotypes under LD growth conditions. Error bars represent SE from three independent biological repeats (n = 8 in each repeat). Different letters indicate significant difference determined by one-way ANOVA test with Bonferroni's correction ($p<0.05$).

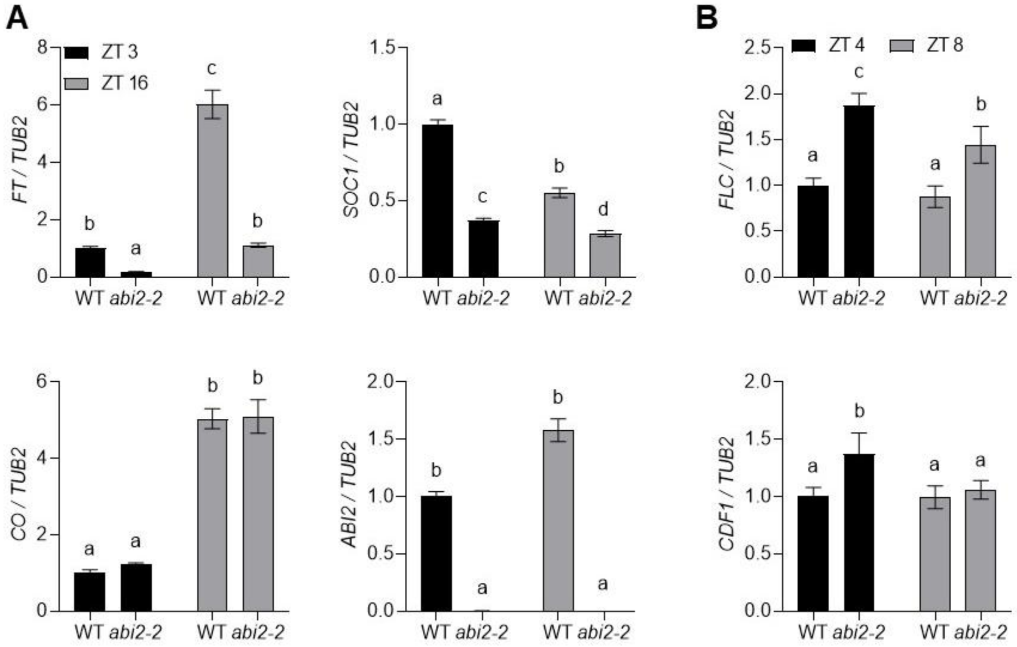

Supplementary Fig. S4 (reference to Fig. 4). Expression of pattern of flowering related genes. (A) Expression analysis of the flowering-time-related marker genes *FT, SOC1* and *CO* in WT and *abi2-2* mutant under LD conditions at different ZT times. Transcript level of *FT* and *SOC1* was significantly decreased in *abi2-2* mutant. *ABI2* was used as experimental control. Transcript level was analyzed by qRT-PCR. *TUB2* was used as internal control. Error bars represent SE from three biological repeats. **(B)** In contrast to FT and SOC1, transcript level of *FLC* and *CDF1* was significantly increased in *abi2-2* mutant. Transcript level was analyzed by qRT-PCR. *TUB2* was used as internal control. Error bars represent SE from three biological repeats. Different letters indicate significant difference determined by one-way ANOVA test with Bonferroni's correction ($p<0.05$). Primers used in the qRT-PCR assay are listed in Supplementary Table S1.

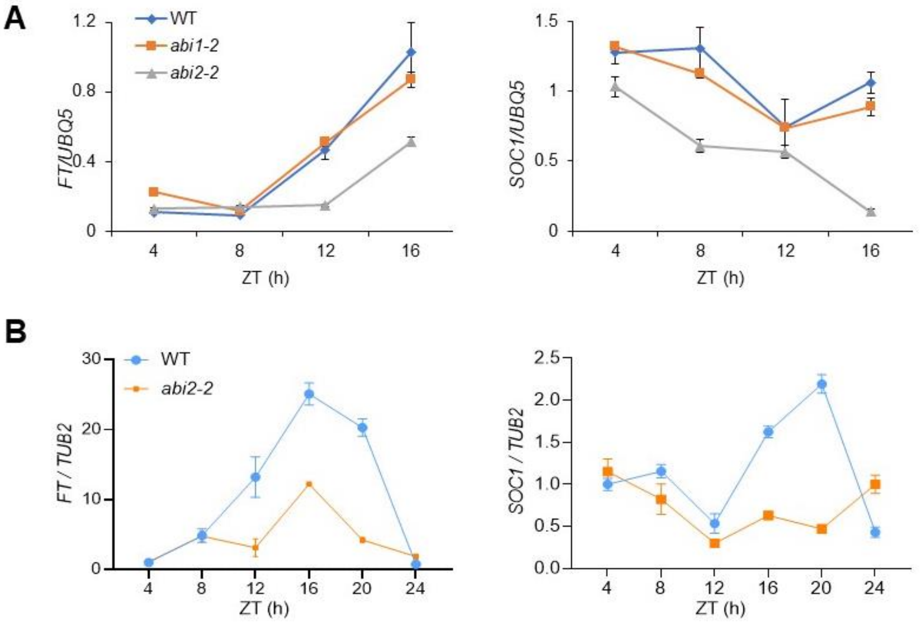

Supplementary Fig. S5. *FT* and *SOC1* were less expressed in *abi2-2* plants. (A) Expression analysis of the flowering-time-related marker genes *FT* and *SOC1* in WT, *abi1-2* and *abi2-2* plants under LD growth conditions at different time points (ZT 4~16). The expression of *FT* and *SOC1* was significantly decreased in *abi2-2* mutant but remains the same in *abi1-2* as that in WT. Transcript level was analyzed by qRT-PCR. *UBQ5* was used as internal control. Error bars represent SE from three biological repeats. **(B)** Expression analysis of *FT* and *SOC1* in WT and *abi2-2* plants under LD growth conditions at different time points (ZT 4~24). The expression of *FT* and *SOC1* was significantly decreased in *abi2-2* mutant compared with WT. Transcript level was analyzed by qRT-PCR. *TUB2* was used as internal control. Error bars represent SE from three biological repeats. Primers used in the qRT-PCR assay are listed in Supplementary Table S1.

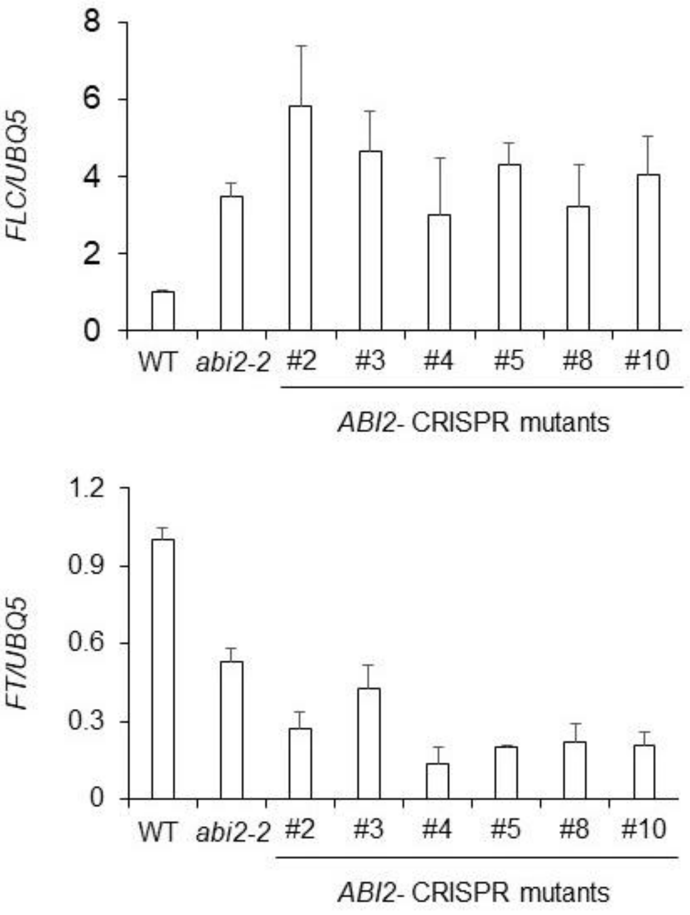

Supplementary Fig. S6. Expression pattern of *FLC* and *FT* in *ABI2-CRISPR* lines. Expression analysis of *FLC* and *FT* in ABI2-CRISPR mutants. *FLC* was highly expressed in *ABI2*-CRISPR lines while *FT* was less expressed compared to WT. Transcript level was analyzed by qRT–PCR. *UBQ5* was used as internal control. Error bars represent SE (with three independent biological replicates). Primers used in the qRT–PCR assay are listed in Supplementary Table S1.

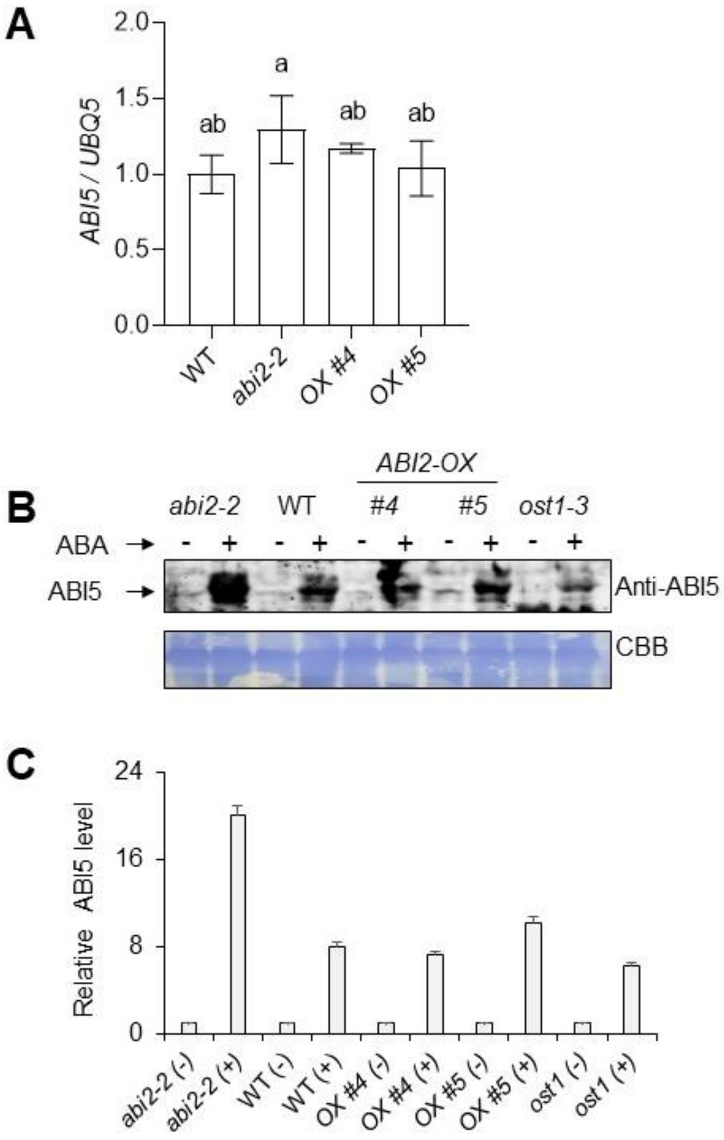

Supplementary Fig. S7. Transcript and protein levels of *ABI5* in *abi2-2* and *ABI2-OX* plants. Transcript and protein abundance of ABI5 was measured in 10-day-old MS plates grown WT, *abi2-2* and *ABI2*-over-expressing lines (OX#4 and OX #5). **(A)** Transcript level of ABI5 slightly increased in *abi2-2* plants compared to WT and ABI2-overexpression plants. Transcript level was analyzed by qRT–PCR. *UBQ5* was used as internal control. Error bars represent SE from three biological repeats. Different letters indicate significant difference determined by one-way ANOVA test with Bonferroni's correction ($p<0.05$). Primers used in the qRT-PCR assay are listed in Supplementary Table S1. **(B)** ABI5 protein accumulates more abundantly in *abi2-2* than in WT. Ten-day-old MS plates grown seedlings of indicated

genotypes were treated without (-) or with (+) ABA (100 μM) for 3 h and then total proteins were extracted. Western blot was carried out using anti-ABI5 antibodies. The experiment was repeated twice with similar results. CBB indicates equal amount of protein loading. **(C)** Relative band intensity of ABI5 in **B**. Error bars represent SE from three biological replicates.

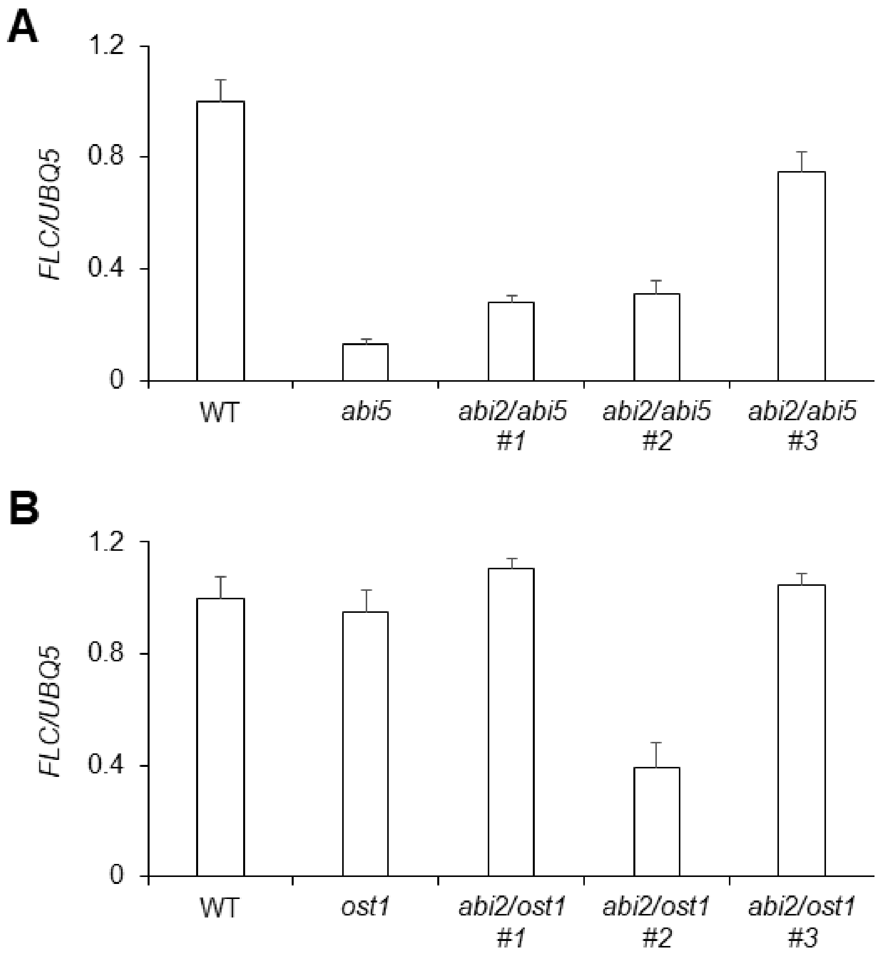

Supplementary Fig. S8. Transcript level of *FLC* in *abi2/ost1* and *abi2/abi5* double mutants. Expression analysis of the *FLC* transcript in WT, *abi5-1*, *abi2/abi5* double mutant **(A)** and in *ost1-3* and *abi2/ost1* double mutant **(B)** grown under LD growth conditions. Transcript level was analyzed by qRT-PCR. *UBQ5* was used as internal control. Error bars represent SE from two biological repeats. Primers used in the qRT-PCR assay are listed in Supplementary Table 1.

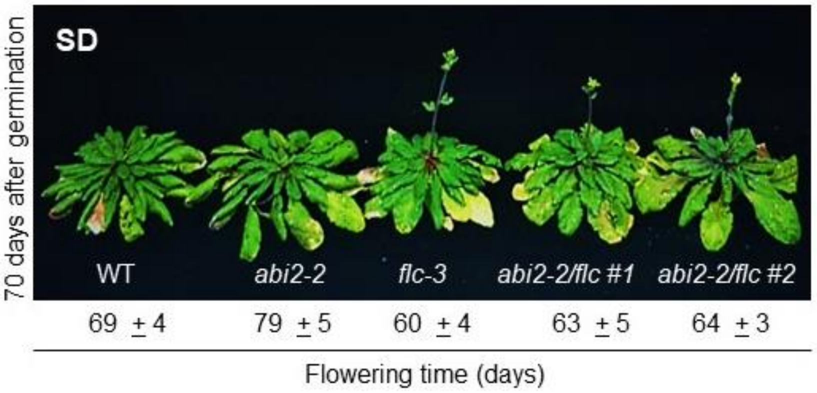

Supplementary Fig. S9. *FLC*-mutation rescues *abi2-2* late flowering phenotypes. Representative (70-day-old) soil grown WT (Col-0), *abi2-2*, *flc-3*, *abi2-2/flc-3 #1* and *abi2-2/flc-3 #1* plants under SD conditions. Flowering time was scored as the number of as the days from germination to flowering of the indicated genotypes under SD growth conditions. ± represents standard errors (from three independent biological repeats (n = 6 in each repeat).

Supplemental Table 1. Primers used in this study.

Primers used for qRT-PCR.

UBQ5-real time-F GACGCTTCATCTCGTCC

UBQ5-real time-R GTAAACGTAGGTGAGTCC

TUB2-real time-F AGCAAATGTGGGACTCCAAG

TUB2-real time-R CACCTTCTTCATCCGCAGTT

ABI2-real time-F TATTTGTCGAAGATGGCTT

ABI2-real time-R TCAATTCAAGGATTTGCT

ABI5-real time-F AACATGCATTGGCGGAGT

ABI5-real time-R TTGTGCCCTTGACTTCAAACT

FLC-real time-F GGCGATAACCTGGTCAAGAT

FLC-real time-R TTTGACTGATGATCCAAGGC

CDF1-real time-F GAGGAATCTCCTCCGTGGCT

CDF1-real time-R GCAAGCTCTCCCCAAGCTCT

FT-real time-F CTGGAACAACCTTTGGCAAT

FT-real time-R AGCCACTCTCCCTCTGACAA

SOC1-real time-F AATTCGCCAGCTCCAATATG

SOC1-real time-R CCTCGATTGAGCATGTTCCT

CO-real time-F ATTCTGCAAACCCACTTGCT

CO-real time-R CCTCCTTGGCATCCTTATCA

Primers used for Cloning.

ABI2-attB1 AAAAAAGCAGGCTTCATGGACGAAGTTTCTCCTGC

ABI2-attB2 AGAAAGCTGGGTCTCAATTCAAGGATTTGCTCT

ABI5-attB1 AAAAAAGCAGGCTTCATGGTAACTAGAGAAACGAAG

ABI5-attB2 AGAAAGCTGGGTCTTAGAGTGGACAACTCGGGTT

sgRNA sequences used for generation of ABI2-CRISPR lines.

sgRNA1. TCCGCCGGAGATGAAATCAA

sgRNA2. CCTTAAACCGTCAGTAATTC

Primers used for genotyping of ABI2-CRISPR lines.

ABI2-FL-For ATGGACGAAGTTTCTCCTGC

ABI2-FL-Rev ATTCAAGGATTTGCTCTTGAA
